# Disparities in COVID-19 Vaccination Coverage Among Health Care Personnel Working in Long-Term Care Facilities, by Job Category, National Healthcare Safety Network — United States, March 2021

**DOI:** 10.15585/mmwr.mm7030a2

**Published:** 2021-07-30

**Authors:** James T. Lee, Sandy P. Althomsons, Hsiu Wu, Daniel S. Budnitz, Elizabeth J. Kalayil, Megan C. Lindley, Cassandra Pingali, Carolyn B. Bridges, Andrew I. Geller, Amy Parker Fiebelkorn, Samuel B. Graitcer, James A. Singleton, Suchita A. Patel

**Affiliations:** ^1^CDC COVID-19 Response Team; ^2^Epidemic Intelligence Service, CDC; ^3^Lantana Consulting Group, Inc., East Thetford, Vermont.

Residents of long-term care facilities (LTCFs) and health care personnel (HCP) working in these facilities are at high risk for COVID-19–associated mortality. As of March 2021, deaths among LTCF residents and HCP have accounted for almost one third (approximately 182,000) of COVID-19–associated deaths in the United States (*1*). Accordingly, LTCF residents and HCP were prioritized for early receipt of COVID-19 vaccination and were targeted for on-site vaccination through the federal Pharmacy Partnership for Long-Term Care Program (*2*). In December 2020, CDC’s National Healthcare Safety Network (NHSN) launched COVID-19 vaccination modules, which allow U.S. LTCFs to voluntarily submit weekly facility-level COVID-19 vaccination data.* CDC analyzed data submitted during March 1–April 4, 2021, to describe COVID-19 vaccination coverage among a convenience sample of HCP working in LTCFs, by job category, and compare HCP vaccination coverage rates with social vulnerability metrics of the surrounding community using zip code tabulation area (zip code area) estimates. Through April 4, 2021, a total of 300 LTCFs nationwide, representing approximately 1.8% of LTCFs enrolled in NHSN, reported that 22,825 (56.8%) of 40,212 HCP completed COVID-19 vaccination.^†^ Vaccination coverage was highest among physicians and advanced practice providers (75.1%) and lowest among nurses (56.7%) and aides (45.6%). Among aides (including certified nursing assistants, nurse aides, medication aides, and medication assistants), coverage was lower in facilities located in zip code areas with higher social vulnerability (social and structural factors associated with adverse health outcomes), corresponding to vaccination disparities present in the wider community (*3*). Additional efforts are needed to improve LTCF immunization policies and practices, build confidence in COVID-19 vaccines, and promote COVID-19 vaccination. CDC and partners have prepared education and training resources to help educate HCP and promote COVID-19 vaccination coverage among LTCF staff members.^§^

LTCFs voluntarily reported HCP COVID-19 vaccination data using the NHSN COVID-19 vaccination modules through April 4, 2021. Coverage was assessed among LTCF HCP, stratified by job category (denominator).^¶^ Vaccinated HCPs (numerator) were those who were vaccinated at the facility or had documentation of receipt of COVID-19 vaccination elsewhere. The module required responses for the total number of HCP and their COVID-19 vaccinations status; subtotals by job categories were optional. Facilities were included for analysis only if they had reported nonzero values for the number of HCP and their vaccination status by every job category in the most recent weekly report submitted through NHSN during March 1–April 4, 2021. Reported HCP job categories were 1) physicians and advanced practice providers (residents, fellows, advanced practice nurses, and physician assistants); 2) therapists (respiratory, occupational, physical, speech, and music therapists, and therapy assistants); 3) ancillary services employees (environmental, laundry, maintenance, and dietary services); 4) nurses (registered nurses and licensed practical/vocational nurses); 5) aides (certified nursing assistants, nurse aides, medication aides, and medication assistants); and 6) other HCP (personnel not included in the preceding categories, including contract staff members, students, and other nonemployees).

Vaccination coverage for aides, the largest HCP category, was further assessed by social indicators within the zip code area of the LTCF, including median income and percentage of adults belonging to racial and ethnic minority groups, percentage living in poverty, and percentage without a high school diploma; social indicator data were obtained from the 2019 American Community Survey.** Tertiles (higher, moderate, and low) were calculated for each indicator based on the national distribution of zip code areas; facilities in the corresponding zip code area were assigned to each tertile. Because this was a convenience sample, with likely intra-facility or locality clustering in vaccination behavior, confidence intervals were not calculated, nor was statistical testing for percentages performed. One LTCF was excluded from this analysis because a corresponding zip code area was missing. Data were downloaded for analysis on April 7, 2021, and all analyses were conducted using SAS statistical software (version 9.4; SAS Institute). This activity was reviewed by CDC and conducted consistent with applicable federal law and CDC policy.^††^

During March 1–April 4, a total of 1,898 LTCFs voluntarily reported HCP COVID-19 vaccination data, including 300 (16%) facilities from 47 states^§§^ that reported numbers for HCP and vaccination status for every job category in the most recent weekly report submitted through NHSN (Supplementary Table 1, https://stacks.cdc.gov/view/cdc/108137). Among 40,212 HCP from these LTCFs, 22,825 (56.8%) had completed COVID-19 vaccination (Table 1). In this convenience sample, the group with the highest percentage of reported fully vaccinated HCP were physicians and other advanced practice providers (75.1%), followed by therapists (69.2%), ancillary services employees (58.5%), nurses (56.7%), and aides (45.6%). Coverage was 68.5% among other HCP not reported in these categories (e.g., students and contractors). The proportion of persons who declined COVID-19 vaccination ranged from 11.1% among physicians to 33.2% among aides. Reported recent COVID-19 infections ranged from 0.7% among physicians to 3.0% among aides. The percentage of aides who were completely vaccinated was lower among those working in facilities located in zip code areas with higher proportions of ethnic and racial minorities (43.5% versus 50.5%), lower household median income (40.5% versus 48.1%), higher poverty (42.4% versus 49.2%), and lower high school completion (42.2% versus 49.3%) (Table 2).

**TABLE 1:** COVID-19 vaccination coverage of health care professionals, by job category, in 300 long-term care facilities reporting to the National Healthcare Safety Network — United States, March 1–April 4, 2021

HCP job category	No. of HCP	No. (%)		
		Fully vaccinated	Declined vaccination	Recent SARS-CoV-2 infection
Aides*	12,670	5,778 (45.6)	4,204 (33.2)	382 (3.0)
Ancillary services employees^†^	9,116	5,337 (58.5)	2,374 (26.0)	172 (1.9)
Nurses^§^	8,622	4,887 (56.7)	2,359 (27.4)	196 (2.3)
Therapists^¶^	3,028	2,095 (69.2)	527 (17.4)	51 (1.7)
Physicians and advanced practice providers**	1,284	964 (75.1)	142 (11.1)	9 (0.7)
Other HCP^††^	5,492	3,764 (68.5)	794 (14.5)	78 (1.4)
**All staff members**	**40,212**	**22,825 (56.8)**	**10,400 (25.9)**	**888 (2.2)**

**Abbreviation**: HCP = health care personnel.

^*^ Certified nursing assistants, nurse aides, medication aides, and medication assistants.

^†^ Environmental, laundry, maintenance, and dietary services.

^§^ Registered nurses and licensed practical/vocational nurses.

^¶^ Respiratory, occupational, physical, speech, and music therapists, and therapy assistants.

** Physicians, residents, fellows, advanced practice nurses, and physician assistants.

^††^ Personnel not included in the preceding categories, including contract staff members, students, and other nonemployees.

**TABLE 2:** COVID-19 vaccination coverage among aides,* by selected social vulnerability metrics and tertile — United States, March 1–April 4, 2021

Social vulnerability metric	Vulnerability tertile, no. vaccinated/total (%)		
	Higher	Moderate	Low
Percentage in racial/ethnic minority group^†^	2,794/6,416 (43.5)	2,379/5,056 (47.1)	605/1,198 (50.5)
Median income^§^	1,245/3,072 (40.5)	1,843/4,005 (46.0)	2,690/5,593 (48.1)
Percentage living in poverty^¶^	1,865/4,397 (42.4)	1,705/3,783 (45.1)	2,208/4,490 (49.2)
Percentage without high school diploma**	1,577/3,739 (42.2)	1,997/4,460 (44.8)	2,204/4,471 (49.3)

* Certified nursing assistants, nurse aides, medication aides, and medication assistants.

^†^ Higher vulnerability tertile: zip code areas with >17.4% persons belonging to racial/ethnic minorities; moderate: 17.4%–96.0%; low: <4.0%.

^§^ Higher vulnerability tertile: zip code areas with household median income ≤$48,770; moderate: median income >$48,770 through ≤64,741; low: median income >$64,741.

^¶^ Higher vulnerability tertile: zip code areas with >15.5% of households living under the federal poverty line; moderate: 15.5%–8.1%; low: ≤8.0%.

** Higher vulnerability tertile: zip code areas with >13.6% of persons aged ≥25 years without a high school diploma or equivalent; moderate: 13.6%–6.9%; low: <6.9%.

## Discussion

In March 2021, data from a convenience sample of 300 LTCFs across the United States indicated disparities in HCP COVID-19 vaccination coverage, with a 30 percentage-point difference in coverage between physicians and other advanced practice providers (75.1%) and aides (45.6%). Among aides, lower vaccination coverage was observed in those facilities located in more socially vulnerable zip code areas. Together, these data suggest that vaccination disparities among job categories likely mirror social disparities in general as well as disparities in the surrounding communities. These findings suggest that vaccination promotion and outreach efforts focused on socially vulnerable and marginalized groups and communities could help address inequities (*4*).

One concern is that nurses and aides in this sample, who have the most patient contact, had the lowest vaccination coverage. COVID-19 outbreaks have occurred in LTCFs in which residents were highly vaccinated, but transmission occurred through unvaccinated staff members (*5*). This finding also has equity implications: national data indicated that aides in nursing homes are disproportionately women and members of racial and ethnic minority groups, with median hourly wages of $13–$15 per hour^¶¶^ (*6*); aides are also more likely to have underlying conditions that put them at risk for adverse outcomes from COVID-19 (*7*). As vaccination was made available on site and lower vaccination rates reflected higher declination rates, vaccine hesitancy might have been an important contributor to undervaccination in these facilities.

The finding that vaccination coverage among aides was lower among those working at LTCFs located in zip code areas with higher social vulnerability is consistent with an earlier analysis of overall county-level vaccination coverage by indices of social vulnerability (*3*); however, similar patterns among LTCF staff members are notable because on-site vaccination removed a number of barriers to vaccination, including travel, scheduling, and need to take time off from work.

The findings in this report are subject to at least five limitations. First, facilities included in this analysis had completed a series of optional fields in a voluntary NHSN COVID-19 vaccination module. The 300 facilities presented represent <2% of the >17,000 LTCFs enrolled in NHSN; thus, the findings from this nonprobability–based convenience sample are not generalizable to all LTCFs. Second, LTCFs reported aggregate weekly data, preventing person-level analysis (e.g., by race/ethnicity) and possibly resulting in duplication of reports, if, for example, HCP work at multiple facilities. Third, data on staff member numbers and numbers vaccinated were self-reported by LTCFs and were not independently validated. Fourth, excluding LTCFs reporting zero values might exclude LTCFs with no vaccine coverage (as opposed to nonreporting), thus inflating the estimated vaccination coverage. Finally, this analysis captured vaccination patterns during March 2021, when most facilities had completed on-site vaccination through the federal pharmacy program. Increasing availability and acceptance of COVID-19 vaccinations in subsequent months might have resulted in higher coverage. However, higher staff member turnover in some job categories, including aides, relative to other job categories, might lead to changes in vaccination coverage.

Low vaccination coverage among LTCF staff members highlights disparities across HCP groups, and in the surrounding communities. Additional efforts are warranted to improve LTCF immunization policies and vaccination practices, build HCP confidence in COVID-19 vaccines, and encourage vaccination among persons who have been economically or socially marginalized. On May 11, 2021 the Centers for Medicare & Medicaid Services (CMS) published an interim final rule requiring LTCFs to educate HCP on COVID-19 vaccines, offer vaccination, and report vaccination status to NHSN*** (*8*). CDC and partners have prepared education and training resources to help educate HCP and promote vaccination coverage among LTCF staff members.^†††^ Finally, LTCFs could consider best practices from influenza campaigns, which found that employer vaccination requirements were associated with the highest vaccination coverage (*9*).

**Summary**

**What is already known about this topic?**

Residents of long-term care facilities (LTCFs) and health care personnel (HCP) who work in these facilities were prioritized for early COVID-19 vaccination. Achieving high coverage in this setting is critical to preventing additional outbreaks.

**What is added by this report?**

During March 2021, 300 LTCFs reported COVID-19 vaccination coverage for their HCP. COVID-19 vaccination coverage was highest among physicians (75.1%) and lowest among aides (45.6%). Vaccination coverage among aides was lower in facilities located in zip code areas with higher levels of social vulnerability.

**What are the implications for public health practice?**

Additional efforts to improve LTCF immunization practices, build confidence in COVID-19 vaccines, and promote COVID-19 vaccination are needed.

## Footnotes

*
https://www.medrxiv.org/content/10.1101/2021.05.14.21257224v1

† Completed COVID-19 vaccination was defined as 2 doses of Pfizer-BioNTech or Moderna or 1 dose of the Janssen (Johnson & Johnson) COVID-19 vaccines. https://www.cdc.gov/vaccines/covid-19/clinical-considerations/covid-19-vaccines-us.html

§
https://www.cdc.gov/vaccines/covid-19/toolkits/long-term-care/index.html

¶
https://www.cdc.gov/nhsn/forms/57.219-p.pdf

**
https://www.census.gov/programs-surveys/acs

†† 45 C.F.R. part 46.102(l)(2), 21 C.F.R. part 56; 42 U.S.C. Sect. 241(d); 5 U.S.C. Sect. 552a; 44 U.S.C. Sect. 3501 et seq.

§§ No LTCFs were included from Delaware, Minnesota, or Montana.

¶¶
https://phinational.org/wp-content/uploads/2020/01/Its-Time-to-Care-2020-PHI.pdf

*** CDC and CMS data are available at the following: https://www.cdc.gov/nhsn/covid19/ltc-vaccination-dashboard.html and https://data.cms.gov/covid-19/covid-19-nursing-home-data

†††
https://www.cdc.gov/vaccines/covid-19/toolkits/long-term-care/index.html
